# Fetal growth restriction induced by maternal gal-3 deficiency is associated with altered gut-placenta axis

**DOI:** 10.1038/s41419-024-06962-6

**Published:** 2024-08-08

**Authors:** Yiran Xie, Fangqi Zhao, Yiru Wang, Sophia Borowski, Nancy Freitag, Irene Tirado-Gonzalez, Naomi Hofsink, Urte Matschl, Torsten Plösch, Mariana G. Garcia, Sandra M. Blois

**Affiliations:** 1 Department of Obstetrics and Fetal Medicine, University Medical Center Hamburg-Eppendorf, Hamburg, Germany; 2 Charité – Universitätsmedizin Berlin, corporate member of Freie Universität Berlin and Humboldt-Universität zu Berlin, and Berlin Institute of Health (BIH) and Institute of Biochemistry, Berlin, Germany and Deutsches Zentrum für Herz-Kreislauf-Forschung (DZHK), partner site Berlin, Berlin, Germany; 3 Charité – Universitätsmedizin Berlin, corporate member of Freie Universität Berlin and Humboldt-Universität zu Berlin, Experimental and Clinical Research Center (ECRC), Berlin, Germany; 4 Institute for Tumor Biology and Experimental Therapy, Georg-Speyer-Haus, Frankfurt, Germany; 5 Department of Obstetrics and Gynaecology, University Medical Center Groningen, University of Groningen, Groningen, The Netherlands; 6 Department Virus Immunology, Heinrich Pette Institute, Leibniz Institute for Experimental Virology, Hamburg, Germany; 7 Perinatal Neurobiology, Department of Human Medicine, School of Medicine and Health Sciences, Carlvon Ossietzky University Oldenburg, Oldenburg, Germany

**Keywords:** Reproductive disorders, Endocrine reproductive disorders

## Abstract

Adverse intrauterine conditions may cause fetal growth restriction (FGR), a pregnancy complication frequently linked to perinatal morbidity and mortality. Although many studies have focused on FGR, the pathophysiological processes underlying this disorder are complex and incompletely understood. We have recently determined that galectin-3 (gal-3), a β-galactoside-binding protein, regulates pregnancy-associated processes, including uterine receptibility, maternal vascular adaptation and placentation. Because gal-3 is expressed at both sides of the maternal-fetal interface, we unraveled the contribution of maternal- and paternal-derived gal-3 on fetal-placental development in the prenatal window and its effects on the post-natal period. Deficiency of maternal gal-3 induced maternal gut microbiome dysbiosis, resulting in a sex-specific fetal growth restriction mainly observed in female fetuses and offspring. In addition, poor placental metabolic adaptions (characterized by decreased trophoblast glycogen content and insulin-like growth factor 2 (*Igf2*) gene hypomethylation) were only associated with a lack of maternal-derived gal-3. Paternal gal-3 deficiency caused compromised vascularization in the placental labyrinth without affecting fetal growth trajectory. Thus, maternal-derived gal-3 may play a key role in fetal-placental development through the gut-placenta axis.

## Introduction

Fetal growth restriction (FGR) is a complex obstetrical complication affecting 3–7% of all pregnancies [1]. This disorder is defined as low fetal birth weight due to reduced growth velocity and a pathological condition where the fetus fails to achieve its intrinsic growth potential [2]. Fetal pathology can increase the risk of neonatal morbidity and mortality with no currently available treatment aside from timely delivery [3]. Hence, prompt recognition and intervention strategies are urgently needed to improve the perinatal outcomes of FGR pregnancies and prevent adverse impacts on children’s quality of life. FGR is etiologically associated with maternal, fetal, or placental factors; however, there is a significant overlapping of pathogenesis [4]. Despite its etiology, poor placenta function is a well-established cause of FGR [5]; therefore, studies aiming to understand the early stages of placental development could reveal the reasons behind pregnancy disorders such as FGR. In this regard, maternal gut microbiota dysbiosis and dysregulated metabolites have been recently associated with the FGR pathogenesis [6], suggesting a critical host-microbiome interaction in placenta insufficiency disease.

Galectin-3 (gal-3) is a member of the β-galactoside-binding galectin protein family, which contains a single carbohydrate recognition domain connected to a N-terminal region. This lectin is the second most abundant galectin at the maternal-fetal interface, where it contributes to endometrial receptivity [7], maternal immune regulation [8], vascular expansion [9, 10], and placenta development [11] (e.g. trophoblast migration and invasion). Healthy pregnancy is associated with upregulation of maternal gal-3 levels during the second and third trimester [10]; however, reduced circulating gal-3 levels characterize pregnancies complicated with FGR. Furthermore, lower placental gal-3 expression is also observed in FGR human pregnancies correlating with placenta dysfunction [12]. Moreover, deficiency of gal-3 during gestation leads to placenta insufficiency and retarded fetal growth in mice [12], suggesting that this lectin is involved in the development of FGR in mammalian pregnancy. However, it has not yet been established whether the placenta failure is dependent on an extrinsic cause (maternal compartment) or is secondary to an intrinsic problem in the placental compartment itself. In this regard, previous research suggested that maternal-derived gal-3 greatly influenced the risk of FGR in mice [12]; however, it remains to be defined which effect of gal-3 deficiency within the maternal compartment contributes to the placenta and subsequent FGR development.

Here, we addressed the maternal compartment response induced by gal-3 deficiency during gestation and its impact on fetal-placental development. We show that lack of maternal gal-3 provokes a dysbiosis of the maternal gut microbiome, favoring the abundance of Bacteroidetes associated with the alternative glucose metabolism during gestation. In addition, maternal gal-3 deficiency resulted in a delayed sex-specific fetal growth trajectory with an asymmetric FGR phenotype. Hypomethylation of placenta Insulin-like growth factor 2 (*IGF2*), which has an important role in fetal growth, was exclusively observed in maternal gal-3 deficient dams. We propose that maternal-derived gal-3 is necessary for proper placental function and fetal growth; dysregulation of this lectin may lead to placenta metabolic defects through maternal gut microbiome dysbiosis and development of FGR.

## Results

### Maternal-derived gal-3 is a key determinant of the female offspring’s growth trajectory

**Fig. 1:**
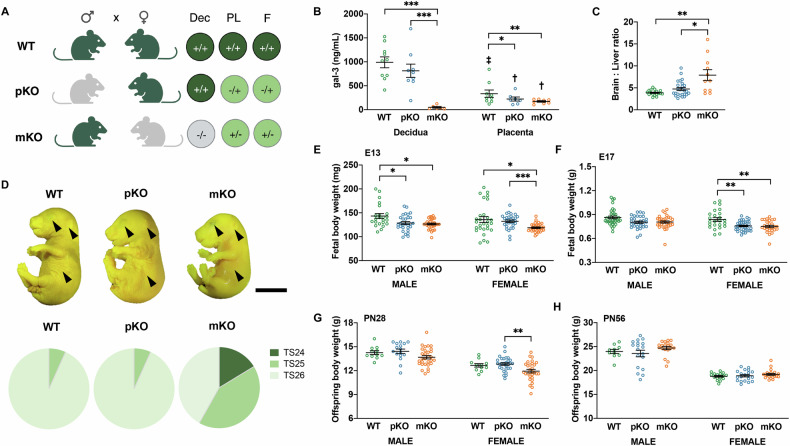
Maternal gal-3 deficiency compromised female fetal/ offspring growth trajectory. **A** Schematic diagram of reciprocal mating to generate the gal-3 wild-type (WT), paternal gal-3 deficiency (pKO) and maternal gal-3 deficiency (mKO). Gal-3 genotype is indicated in the decidua (Dec), placenta (PL) and fetus (F) during pregnancy. **B** Gal-3 expression levels examined by ELISA in the decidua and placenta on embryonic day (E)13 (*n* = 8–10). **C** Brain-to-liver weight ratio on E13, fetuses carried by gal-3 mKO dams exhibited asymmetric fetal growth restriction (FGR) (*n* = 11–26). **D** Theiler stage (TS) analysis was conducted to assess the fetal development on E17. As shown in the representative photos, fetuses carried by gal-3 mKO dams showed visible eyes through the eyelids (upper arrow), uncovered ear canal (middle arrow) and fewer skin wrinkles (lower arrow) (upper panel, scale bar = 0.50 cm). Additionally, the majority of gal-3 mKO fetuses only reached TS24–25, suggesting delayed fetal development compared to WT and pKO fetuses (lower panel, *n* = 38–45). **E**, **F** Body weights of male and female fetuses on E13 and E17 respectively. **G**, **H** Sex-specific offspring body weight at post-natal day (PN)28 and PN56 respectively. All data were presented as the mean ± SEM. *P < 0.05, **P < 0.01 and ***P < 0.001 using one-way ANOVA followed by Tukey’s multiple comparisons test or Kruskal–Wallis test followed by Dunn’s multiple comparisons test. ^†^*P* < 0.01 and ^‡^*P* < 0.001 using unpaired Student’s *t*-test or Mann–Whitney *U*-test to assess the decidual and placental gal-3 expression levels within each group.

To investigate the contribution of gal-3 from different parental origin on placental and fetal development, reciprocal mating was used to achieve gal-3 deficiency in either the paternal (pKO) or maternal (mKO) compartment (Fig. 1A). First, we examined the gal-3 expression levels at the feto-maternal interface during the post-placentation period. As depicted in Fig. 1B, in both WT and pKO dams, gal-3 levels were significantly higher in the maternal decidua on E13 than in the placental compartment, whereas increased gal-3 in the placenta compared with decidua was observed in mKO. Because maternal gal-3 deficiency leads to increased fetal demise and FGR [12], we further characterized fetal growth based on additional fetal biometric parameters. Thus, fetuses derived from gal-3 mKO dams displayed asymmetric FGR characterized by a higher brain-to-liver weight ratio (Fig. 1C). Since FGR is often accompanied by fetal development retardation in mice [13], we conducted Theiler stage (TS) analysis to evaluate the development trajectory in fetuses carried by mKO or pKO dams. As noted, fetuses born to gal-3 mKO dams exhibited a delayed fetal development on E13 [12]. As shown in Fig. 1D, many fetuses carried by gal-3 mKO dams on E17 persistently showed immature fetal developmental stage growth. For instance, most gal-3 WT and pKO fetuses reached TS26, whereas gal-3 mKO fetuses remained at TS24–25 (*P* < 0.001). Considering the potential impact of fetal gender on pregnancy outcomes, we compared the growth trajectories between male and female fetuses at prenatal and post-natal (PN) periods. As depicted in Fig. 1E, male and female fetuses derived from gal-3 mKO dams displayed lower body weight on E13. Interestingly, the variances among different dams were more significant when carrying female fetuses. During late gestation (E17), only female fetuses of gal-3 pKO and mKO dams displayed significantly reduced body weight when compared to the WT counterparts (Fig. 1F). To assess further the short- and long-term effects of gal-3 deficiency on offspring growth, we extended the analysis to PN28 and PN56. Here, we observed that only female offspring derived from gal-3 mKO dams showed a significantly reduced body weight compared to the pKO groups (Fig. 1G). However, this difference disappeared when it progressed to PN56 (Fig. 1H). These findings emphasize the unique role of maternal-derived gal-3 on fetal growth and the impact on female gender fetuses and offspring.

### Gal-3 deficiency in the maternal compartment impacts the placental metabolic and developmental status

**Fig. 2:**
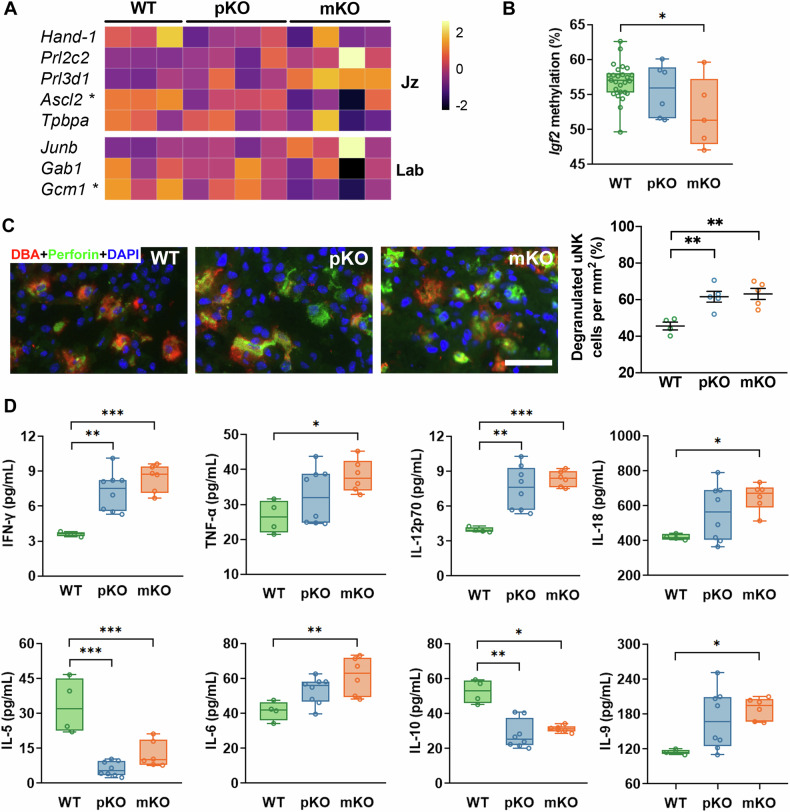
Absence of maternal gal-3 induces placental insufficiency and inflammation state. **A** The relative mRNA expression levels of genes involved in trophoblast differentiation on E13 placentas were visualized as a heatmap (*n* = 3–4). **B** Methylation level of *Igf2* was significantly reduced in the placentas of gal-3 mKO dams (*n* = 5–26). **C** *Dolichos biflorus* agglutinin (DBA) lectin and perforin double fluorescent staining images (scale bar = 50 μm). The percentage of degranulated uNK cells was calculated by dividing the number of uNK cells surrounded by perforin staining but not overlapped (denoted as degranulated uNK cells) by the total number of uNK cells per mm^2^ (*n* = 4–5). **D** Cytokine profile of E13 placentas was analyzed using Luminex technology (*n* = 4–6). Data were presented as box plots. *P < 0.05, **P < 0.01, and ***P < 0.001 using one-way ANOVA followed by Tukey’s multiple comparisons test or Kruskal–Wallis test followed by Dunn’s multiple comparisons test.

Since growth defect is linked to impaired placentation, we next examined the expression levels of genes involved in trophoblast differentiation on E13 placentas. As shown in Fig. 2A and Fig. S2A, *Ascl2*, a spongiotrophoblast marker and an indicator of glycogen storage in the junctional zone (Jz) and *Gcm1*, a transcription factor that facilitates nutrient exchange during labyrinth (Lab) formation, were significantly reduced in the gal-3 mKO dams compared to the WT counterpart. Thus, altered expression levels of trophoblast markers in gal-3 mKO placentas suggest a retarded trophoblast differentiation in the Jz and Lab layers of the placenta. As shown previously, reduced glycogen storage in the Jz was observed in the placenta carried by gal-3 mKO dams [12]. Consistent with this, we observed a significantly decreased methylation level of the *Igf2*, a well-characterized imprinted gene associated with glycogen content and fetal growth [14], in gal-3 mKO placentas compared to the WT controls (Fig. 2B), suggesting that maternal gal-3 is a determinant of the placenta metabolic capacity. When analyzing the fetal vasculature in the Lab using Isolectin B4 (IB4) staining (Fig. S1A), we observed increased vascular branching (Fig. S1B) and vessel density (Fig. S1C); however, Lab lacunarity was significantly reduced in both gal-3 deficiency models (Fig. S1D). These results suggest that reduced blood spaces and less efficiency in nutrients and oxygen exchange are likely associated with an intrinsic disadvantage of gal-3 deficiency within the placental compartment. Notably, we observed significantly reduced methylation levels of *Vegf* only in pKO dams (Fig. S1E).

Inflammation plays a role in the pathophysiology of FGR [15, 16]; therefore we analyzed the activation of uterine natural killer cells (uNK) on E13 using the *Dolichos biflorus* lectin (DBA) lectin (uNK cell marker) and perforin (cytotoxic granules) staining. We observed a higher proportion of degranulated NK cells in the decidua of gal-3 pKO and mKO dams (Fig. 2C). Thus, the absence of gal-3, particularly maternal gal-3, triggered aberrant activation of uNK cells in the maternal decidua, potentially inducing placental inflammation. We next analyzed the placental inflammatory state by Luminex assay to confirm our findings. Interestingly, maternal gal-3 deficiency appeared to increase placenta pro-inflammatory cytokines (such as IFN-gamma, TNF alpha, IL-12p70, IL-18, IL-6, and IL-9) and attenuate anti-inflammatory cytokines (e.g., IL-5 and IL-10) (Fig. 2D; Fig. S2B). Finally, we characterized the galectin signature at the maternal-fetal interface. As depicted in Fig. S1F, a decreased decidual gal-1 expression in the gal-3 pKO and mKO, but we noted a similar expression in the placental compartments. Interestingly, the gal-7 levels were increased in the gal-3 mKO placentas (Fig. S1G). Our findings indicate that the placental function is greatly influenced by maternal-derived gal-3, and the lack of this lectin within the maternal compartment results in a placental inflammatory milieu with a distinguished galectin signature.

### Maternal gut dysbiosis is associated with maternal gal-3 deficiency and FGR outcome

**Fig. 3:**
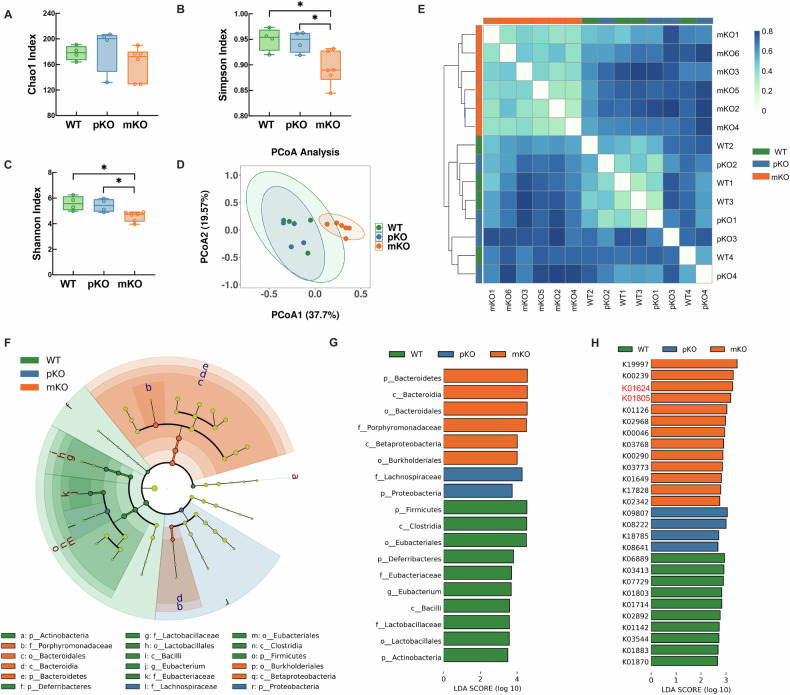
Characterization of the gut microbiota in maternal gal-3 deficient dams. **A** Box and whisker plots depicted alpha diversity of gut microbiome using Chao1 index suggested no significant differences in species richness among gal-3 WT, pKO and mKO dams. **B** Simpson index indicated significantly decreased evenness of gut microbiota in gal-3 mKO dams. **C** Shannon index showed reduced richness and evenness of maternal gut microbiome. **D** Principal component analysis (PCoA) of the gut microbiota from gal-3 WT, pKO and mKO dams. Plots based on bacterial relative abundance profiles displayed clear separation between gal-3 mKO to the other two groups. **E** Bray–Curtis Distance Matrix of the gut microbiota from gal-3 WT, pKO, and mKO dams. **F** Cladogram of the linear discriminant analysis effect size (LEfSe) results on gut microbiota taxa of gal-3 WT, pKO, and mKO dams. Levels of the cladogram represented, from the outer to inner rings: phylum (p_), class (c_), order (o_), family (f_) and genus (g_). **G** Bar plots indicated the differentially abundant bacterial taxa among three groups (LDA ≥ 2). **H** Enriched KEGG orthology terms in the three compared groups (LDA ≥ 2). LDA linear discriminant analysis. Data shown in box and whisker plots were presented as minimum, lower quartile, median, upper quartile and maximum. *P < *0.05*, **P < *0.01* and ***P < *0.001* using one-way ANOVA followed by Tukey’s multiple comparisons test or Kruskal–Wallis test followed by Dunn’s multiple comparisons test.

Recently, several studies have established a connection between maternal gut microbiota and pregnancy complications, including FGR in humans [6, 17, 18]. As fetuses of gal-3 mKO dams displayed asymmetric FGR, we next aimed to characterize the maternal gut microbiota in dams with different gal-3 expression levels in the maternal compartment. According to the Chao1 index (Fig. 3A), the alpha-diversity of the microbiome showed no significant differences in species richness between gal-3 WT, pKO, and mKO dams. Nevertheless, the Simpson index (evenness) and Shannon index (richness and evenness) demonstrated a significant divergence among groups (Fig. 3B, C). The beta-diversity analysis using the principal coordinate analysis (PCoA) revealed a clear difference of maternal gut microbiota among different groups (*P* < 0.001), with 37.70% and 19.57% observed variations represented by principal component (PC)1 and PC2, respectively (Fig. 3D). Likewise, Bray–Curtis dissimilarity displayed two revealed clusters, clearly distinguishing the gal-3 mKO dams from the other two groups (Fig. 3E). Next, we utilized the linear discriminant analysis effect size (LEfSe) method to identify differences in the relative abundance of bacterial taxa across all samples (Fig. 3F). According to the cladogram plot, at the phylum level, Bacteroidetes was highly abundant in the gal-3 mKO dams. In contrast, Firmicutes was dominant in the WT dams (LDA ≥ 2, Fig. 3G). Bacteroidetes, as the most prevalent gram-negative gut bacterial species, is equipped with lipopolysaccharide (LPS) on the outer membrane, which is responsible for microbial-host interaction [19]. Interestingly, LPS can induce FGR in mice due to inflammation and oxidative stress [20]. Also, gal-3 can interact with LPS through its N′ and C′ terminal regions, negatively regulating LPS-mediated inflammation, which protects the host from endotoxin shock [21]. Based on these findings, we hypothesized that maternal gal-3 deficiency gut microbiota dysbiosis (higher abundance of gram-negative Bacteroidetes) may contribute to LPS-induced inflammation and FGR progression. To test this hypothesis, pregnant gal-3 WT and KO dams were injected with 2.5 μg LPS intraperitoneally (i.p.) on E7 and then sacrificed on E13 (Fig. S1H). We found that gal-3 KO dams were more prone to fetal demise in response to LPS in comparison to the gal-3 WT dams (Fig. S1I and S1J), indicating that gal-3 plays a protective role against the LPS-induced fetal demise.

Finally, we performed KEGG orthology enrichment analysis to gain a deeper insight into the function of the maternal gut microbiome. We detected thirteen significantly enriched KEGG orthology terms in the gut microbiota of gal-3 mKO dams (LDA ≥ 2, Fig. 3H). Additionally, as depicted in Fig. S3, several enriched KEGG orthology terms in gal-3 mKO dams were found to be upregulated in multiple pathways of fructose and mannose metabolism (marked in red). It is worth noting that in some glucose-depleted cases, fructose can serve as an alternative source to fulfill the increasing energy demand [22]. In line with this, we observed a significant reduction in maternal glucose circulating levels in gal-3 mKO dams on E13 (Fig. S1K). Together, these results suggest that maternal gal-3 deficiency alters the energy metabolism in the maternal gut microbiota.

## Discussion

Intrauterine growth and development are highly dependent on an adequate placenta function. The suboptimal maternal environment is linked to poor placental homeostasis and fetal development, increasing the risk of pregnancy disorders and fetal growth restriction. The heterogeneous character of FGR represents a challenge in research as multiple factors derived from the maternal, placental, and fetal compartments might co-exist and interact with each other [4, 23]. Research in this area addressed the importance of placental response against maternal environmental perturbations in fetal growth [24]. Nevertheless, due to the difficulty in the compartmentalized study of the maternal-fetal interface, the mechanisms by which the maternal environment may lead to FGR are poorly understood. In this study, we supplied evidence that an adverse maternal environment (dictated by the lack of gal-3) impacts female fetal growth by altering the metabolic placental function. In addition, our work highlighted the role of the maternal gut-placental axis in gal-3 deficiency-induced fetal growth restriction. These results demonstrated the significance of gal-3 within the maternal environment and the orchestration of uneventful gestation.

We previously reported that lack of gal-3 in the maternal/ placental compartments caused a systemic pro-inflammatory response, and an aberrant uNK cell decidual infiltration and activation, which compromise placental functions and fetal growth [12]. This work showed that gal-3 is more abundant in the maternal decidua than in the placenta during murine gestation, which is consistent with the phenotypical data from human studies [25, 26], suggesting its role in pregnancy orchestration. Indeed, heterozygous genotype (*Lgals3*^+/−^) fetuses carried by gal-3 pKO dams and mKO dams showed different growth trajectories, which might be attributed to the distinct abundance of maternal-derived gal-3 throughout intrauterine development. Recently, evidence suggested an association between fetal gender and pregnancy outcomes, possibly due to the difference in sex chromosome genes and hormones [27–29]. Interestingly female fetuses/ offspring derived from mKO dams displayed a reduced growth trajectory, which agrees with human studies showing that female gender is an independent risk factor of FGR [30–34]. Regarding gal-3 expression within the placental compartment, Hutter et al. reported minor sex-specific differences in female placental expression compared to male-derived placenta during normal pregnancy. However, FGR cases did not show significant gender-specific differences, with a low expression of gal-3 [35]. Therefore, we propose that maternal-derived gal-3 deficiency independently compromised fetal/ offspring’s growth in a sex-specific manner.

Asymmetric FGR indicates that fetal malnutrition is mainly caused by inadequate placenta function [36, 37]. This study showed that maternal gal-3 deficiency affected the expression levels of *Ascl2* and *Gcm1* genes within the placenta. As a member of the basic helix-loop-helix (bHLH) transcription factor family, *Ascl2* is responsible for the differentiation of trophoblasts to form the junctional zone layer of the placenta where the glycogen is stored as an energy source for the growing conceptus [38, 39]. In line with this, we previously observed a reduction of glycogen trophoblast cells in gal-3 mKO placenta [12], which is associated with this layer’s compromised endocrine capacity e.g. inducing a reduced production of placental hormones, particularly Igf2 [39, 40]. Indeed, we observed a significantly hypomethylation level of *Igf2* in the gal-3 mKO dams, suggesting an impaired energy supply affecting fetal growth. Animal studies suggested that selective deletion of placental-specific *Igf2* in mice results in severe stunting in fetal development due to a decrease in placental glycogen [40, 41]. Besides, hypomethylation of the *Igf2* gene has been reported in the placenta of FGR pregnancies, suggesting the contribution of epigenetic modification to placental and fetal development [42–44]. On the other hand, the vascularization of the Lab layer also contributes to the normal placental function as it enables the nutrient exchange between mother and fetus [45]. Despite the current results suggesting a decreased *Gcm1* expression in gal-3 mKO placenta, we found that the vessel lacunarity was significantly reduced in both gal-3 deficient dams. In addition, the methylation level of the angiogenic gene *Vegf* was significantly decreased only in gal-3 pKO placenta. Taken together, we postulate that paternal-derived gal-3 mainly influences the Lab layer’s structural and functional integrity, ensuring proper placental perfusion. Consistent with fetal phenotypes reported in this study, the epigenetic dysregulation of the *Vegf* and Lab layer functionality in pKO-derived placenta does not appear to be sufficient to induce FGR, suggesting once again that paternal-derived gal-3 has a minor impact on proper intrauterine fetal growth.

Galectin-3 is expressed on uNK cells and regulates the decidualization and maternal immune response during early pregnancy [46–48]. Although uNK cells are equipped with cytoplasmic granules containing perforin and granzyme, uNK cells are less cytotoxic compared to peripheral NK cells, which contributes to maternal immune adaption and tolerance during pregnancy [49]. In vitro experiments showed that inhibition of gal-3 increased the number of degranulating NK cells, suggesting the involvement of this lectin in NK cell cytotoxicity [50]. Interestingly, we showed that uNK cells undergo functional changes (perforin-releasing) in response to gal-3 deficiency within the maternal compartment. Indeed, uNK cells can be activated abnormally in adverse maternal microenvironments as foes to pregnancy via mediating the cytolysis process [51] and placenta inflammation. Therefore, it is unsurprising that an imbalance of the placental pro-inflammatory/ anti-inflammatory cytokine profile characterized the gal-3 mKO dams during the post-placentation period. Furthermore, an increased placental gal-7 expression on mKO dams will impact the inflammation state. In this context, gal-7 administration in vivo during pregnancy caused a pro-inflammatory placental state (elevated IL-1ß, IL-6, and reduced IL-10), impairing the placentation process in mice [52]. While we found no effect on placental gal-1 expression, the decidual expression of gal-1 was reduced in the mKO dams. Overall, deficiency of maternal gal-3 provokes a shit to an inflammatory galectin signature.

The concept of “gut microbiota-placenta axis” [53–55] suggests that maternal gut microbiota translocating in the feto-maternal interface affects fetal development and offspring health [56, 57]. Apart from microbial colonization of the fetus, several maternal microbiota-derived compounds, such as LPS are recognized by toll-like receptors in innate immune cells at the feto-maternal interface [55]. Galectins often act as pattern recognition receptors and trigger innate immune responses through specific galectin-glycan binding circuits [58]. In particular, gal-3 is the first galectin family member shown to engage glycans from multiple bacterial species and exert antimicrobial activity [59]. The proteolytic processing of gal-3 regulated by meprin metalloproteases maintains mucosal barrier properties, contributing to host-microbiome homeostasis [60]. Notably, we found that the gut microbiota profile of gal-3 mKO dams radically differed from that of WT and pKO dams. Indeed, Bacteroidetes and Firmicutes are the most abundant species in human and mouse intestines [61, 62], and evidence indicate that in healthy mice, the ratio of Firmicutes to Bacteroidetes is increased [63]. Moreover, Firmicutes are high-energy harvesters and might be important for energy storage and fetal growth [64, 65]. In line with this, our results demonstrated that Firmicutes is more abundant in WT dams, whereas Bacteroidetes was significantly higher in the mKO group, which agrees with the findings in pregnant women with FGR [6, 17]. Gal-3 binds and negatively regulates the function of LPS, which is the main component of the outer membrane of Gram-negative Bacteroidetes [21]. Moreover, gal-3 deficient mice excessively produce pro-inflammatory cytokines and are more susceptible to LPS-induced endotoxic shock [21], which aligns with our finding of poor response against LPS-induced fetal demise in gal-3 deficient dams. More importantly, the integrated microbiome and metabolomics research has illustrated the link between microbial signatures and clinical features of pregnancy with FGR [6]. Strikingly, the differential enriched functional categories in FGR pregnancy are reported to be mainly involved in glycometabolism [17]. Interestingly, the gut microbiota of gal-3 mKO dams with asymmetric FGR features demonstrated upregulated fructose metabolism. Since fructose can serve as an alternative source to fulfill the increasing energy demand in a glucose-depleted scenario [22], the reduced placental glycogen storage and the lower glucose circulating levels in gal-3 mKO dams suggest that the enhanced fructose metabolism was insufficient to compensate for the compromised energy supply induced by maternal gal-3 deficiency which adversely affected fetal growth. Despite this, the maternal microbiome analysis also demonstrated that dysbiosis (abundance of Bacteroides) likely contributes to the pro-inflammatory placental milieu and impacts intrauterine growth.

In conclusion, our work emphasizes the individual contribution of maternal-derived gal-3 on normal placental development and its unique role in mediating maternal gut microbial homeostasis, providing novel insight into the implication of dysregulated maternal gal-3 in FGR pathogenesis. These observations provide novel directions for galectinology research at the maternal-fetal interface.

## Materials and methods

### Ethics approval for mouse experiments and tissue collection

The current study was carried out using C57BL/6 mouse strains from Jackson Laboratory. All animal experimental procedures complied with the institutional guidelines of Charité (G0036/13) and University Medical Center Hamburg-Eppendorf (ORG_1082), adhering to German Animal Welfare legislation. The reciprocal mating of gal-3 wild-type (*Lgals3*^+/+^) mice and gal-3 knock-out (*Lgals3*^−/−^) mice was carried out to achieve the paternal (pKO) or maternal (mKO) gal-3 deficiency as previously described [12]. After mating, the identification of a vaginal plug was recorded as embryonic day (E) 0. Pregnant mice were euthanized on E13 or E17, and the whole implantation sites were collected for further analysis. Decidua and placenta tissues separated from the entire implantation sites were frozen or fixed in paraffin for histological sectioning. Embryos obtained on E13/E17 were weighted, and their tails were clipped for DNA extraction and sex determination PCR as previously described [66]. Fetal developmental analysis was conducted after Bouin’s solution fixation according to Theiler Stage criteria [67]. Brain and liver tissues from E13 embryos were separated and weighed to calculate the brain-to-liver weight ratio for assessing FGR type. To evaluate the short- and long-term impacts of gal-3 deficiency, the body weight of offspring derived from different dams was recorded on post-natal day (PN)28 and PN56.

### Enzyme-linked immunosorbent assay (ELISAs)

The decidua and placenta tissues collected on E13 were homogenized mechanically using previously described protocols [68]. Protein concentration from the tissue homogenates was determined using the Bradford assay. Tissue galectins levels were assessed using the mouse gal-3 Duoset ELISA kit (R&D Systems, DY1197), mouse gal-1 ELISA kit (R&D Systems; DY1245), and gal-7 ELISA kit (R&D Systems; DY1304) following the manufacturer’s instructions. Comprehensive protocols are provided in Supplementary information.

### RNA isolation and quantitative real-time PCR

The experimental protocols for total RNA isolation, cDNA generation, and PCR reaction profiles were previously described [12]. The primers utilized are shown in Table S1. The relative gene expression was calculated as 2^-ΔCt^, where ΔCt = Ct _target gene_ − Ct _reference gene_. Results were normalized by Z-score and visualized using a heatmap.

### Epigenetic analysis

The AllPrep DNA/RNA Mini Kit (Qiagen, 80204) was used to extract genomic DNA from E13 placenta lysates following the manufacturer’s protocol. Bisulfite conversion was performed with 500 ng genomic DNA using the EZ DNA methylation-Gold kit (Zymo Research, D5005) according to the instructions provided by the manufacturer. The universal primer approach was used for the gene-specific assays. The primers and conditions for the pyrosequencing of universal biotinylated gene-specific promoters are shown in Table S2. The Pyromark Q24 system and software (Qiagen) were utilized for the pyrosequencing reaction and quantification of the methylation percentage of the individual CpG site. The average methylation levels of each gene were calculated using the individual methylation levels for all measured CpG sites.

### Histological analysis

Cryosections from E13 placentas were prepared at a thickness of 8 μm and stained with Dolichos biflorus agglutinin (DBA) and perforin as described previously [68]. The high-resolution digital slide scanner (Pannoramic MIDI II, 3DHISTECH) was utilized to scan the multiplex fluorescent-stained slides. Captured images were analyzed blindly by two investigators. The degranulated uNK cells were defined as the uNK cells (DBA^+^) surrounded by perforin staining extracellularly (without overlapping with the uNK cells).

### T helper (Th) cytokines profile analysis (Luminex)

Cytokine levels were assessed in the E13 placenta homogenates with the Mouse Th1/Th2/Th9/Th17/Th22/Treg Cytokine 17-Plex ProcartaPlex Panel (Invitrogen, EPX170-26087-901) using the Luminex xMAP (multianalyte profiling) technology and analyzed with the Bio-Plex 200 instrument (Bio-Rad). The protein concentration normalized results and Z-score were calculated to plot the heatmap.

### Statistical analysis

GraphPad Prism (GraphPad Software, version 9.4.1) and SPSS (IBM SPSS Statistics, version 26.0) were utilized for performing the statistical analysis. Bar charts, pie charts, and heatmaps were generated with GraphPad Prism. Data are displayed as mean ± SEM and analyzed using Mann–Whitney *U*-test, one-way ANOVA followed by Tukey’s multiple comparison test or Kruskal–Wallis test followed by Dunn’s multiple comparison test as appropriate. In all analyses, statistical significance was determined if *P* < 0.05.

### Supplementary information

Suppl. Material

## Data Availability

We declare that the data supporting the findings of this study are available within the paper and from the authors upon request.
