# Supplementary material for: Fetal growth restriction induced by maternal gal-3 deficiency is associated with altered gut-placenta axis
Source: Cell Death Dis. 2024 Aug 8;15(8):575. doi: 10.1038/s41419-024-06962-6 (PMC11310209; doi:10.1038/s41419-024-06962-6)
Supplement: Supplementary file 1 — Suppl. Material [file 41419_2024_6962_MOESM1_ESM.docx]

**Supplementary Information**

**Materials and methods**

**Isolectin B4 staining and vasculature analysis**

To visualize the vasculature network of the labyrinth (Lab) in E13 placentas, immunohistochemistry (IHC) staining of Isolectin B4 (IB4) was performed according to the following procedures: Paraffin sections of 8 μm thickness were rinsed in TBS, then exposed to 3% hydrogen peroxidate (H_2_O_2_) for 30 min at room temperature (RT). Slides were then treated with 2% normal serum for 20 min at RT to prevent non-specific binding. Afterwards, the primary antibody against IB4 (diluted 1:200, Sigma-Aldrich, L2140) was incubated overnight at 4 °C. After washing, slides were incubated with the HRP-conjugated secondary antibody (diluted 1:200, Jackson ImmunoResearch, 111-035-047) for 1 h at RT. The 3,3'-diaminobenzidine tetrahydrochloride (DAB, Dako Cytomation) was used as a chromogen to visualize the reaction. Subsequently, slides were washed sufficiently and counterstained with 0.1% Mayer's hematoxylin. Dehydration, transparence, and mounting were performed with routine procedures.

Stained sections were scanned with a high-resolution slide scanner (Pannoramic MIDI II, 3DHISTECH). For each section, 10-15 regions were randomly selected at 20x magnification and converted to invert grayscale pictures for vasculature analysis using AngioTool^1^. Program was set up as follows: vessel diameter and intensity: 7; threshold: 40-255. All images were calibrated using the relationship between pixels and μm. The main outcome measures include branching index (junctions), vessel density and mean lacunarity.

**Murine galectins ELISA**

Concentration of gal-3, gal-1 and gal-7 in the lysates of decidua and placenta were determined with the mouse gal-3 ELISA kit (R&D Systems; DY1197), gal-1 ELISA kit (R&D Systems; DY1245) and gal-7 ELISA kit (R&D Systems; DY1304) respectively. Briefly, 96-well high binding half-area plates (Corning, CLS3690) were coated with the corresponding capture antibodies overnight. After washing 3 times with washing buffer (0.05% Tween-20 in PBS), all wells were blocked with reagent diluent (1% BSA in PBS) for 1 h at RT. Meanwhile, the serial standards were prepared according to the manufacturer’s protocols (1000 to 15.6 pg/mL for gal-3; from 8000 to 125 pg/mL for gal-1; from 6000 to 93.8 pg/mL for gal-7), and lysates were diluted to different concentrations (1/5000 for gal-3; 1/2000 for gal-1; 1/5 for gal-7). After washing 3 times with washing buffer, the corresponding standards and diluted samples were applied in the plate for 2h incubation. Plates were then washed 3 times and incubated 2h with the corresponding detection antibodies at RT. Following that, plates were washed and subsequently incubated with streptavidin-horseradish peroxidase (HRP) for 20 min. After sufficient washing, the solution of 3,3,5,5´-teramethylbenzidine (TMB) substrate was added and incubated for around 5 min in the dark. The reaction was stopped with 4N H_2_SO_4_, and the absorbance at 450 nm was recorded with a microplate reader (Multiskan Sky, Thermo Scientific). The expression levels were extrapolated from the corresponding standard curves using a four-parameter logistic (4-PL) curve fit and normalized to the equal protein concentration.

**Lipopolysaccharide (LPS) treatment**

In a separate animal experiment, pregnant gal-3 WT (*Lgals3*^+/+^) and gal-3 KO (*Lgals3*^-/-^) dams were injected intraperitoneally (i.p.) with 2.5 μg LPS (Escherichia coli LPS O55:B5, Sigma Co.) on E7 (n=4/group). After euthanizing the pregnant mice on E13, the whole implantation sites were collected, and the abortion rate was calculated by dividing the number of fetal resorptions by the total implantations numbers.

**Glucose assay**

The Mouse Glucose Assay (Crystal Chem, 81692) was used to measure the serum glucose level on E13. Reagents and standards (ranging from 250 to 31.25 mg/dL) were prepared according to the manufacturer’s protocol. Briefly, 150 μL enzyme solution was added to a 96-well half-area plate (Corning, CLS3690). Then 1 μL serum samples or standards were applied in the plate and mixed thoroughly. Following a 5-min incubation at 37 °C, the absorbance at 505 nm was recorded by the ELISA plate reader (Multiskan Sky, Thermo Scientific). The ultimate concentration was extrapolated from the standard curves with a linear fit and normalized by the protein concentration of each sample.

**Microbiome analysis**

Fecal samples of pregnant mice were collected on E13 and placed in individual barcoded tubes containing DNA stabilization buffer. DNA extraction, library preparation, and sequencing were conducted with the assistance of Transnetyx (Cordova, TN, USA). Briefly, the extraction of fecal DNA was conducted using a DNeasy 96 PowerSoil Pro QIAcube HT kit (Qiagen, 47021) according to the manufacturer’s instructions. The NanoDrop 2000 Spectrophotometer (Thermo Fisher Scientific) was applied to assess the concentration and purity of extracted DNA for converting into the sequencing library using the KAPA HyperPlus library Prep Kit (KAPA Biosystems). After quality control, the libraries were sequenced by a shotgun sequencing method (2x150 bp, 2 million reads) using the Illumina NovaSeq instrument. Results were encoded in a test-based FASTQ format to allow the storage of both the biological sequences and corresponding quality scores. Taxonomic classification and further analysis were conducted through One Codex Database (One Codex, USA). A k-mer based classification method was introduced to map the processed reads to a microbial reference database^2,3^. The sequencing depth and coverage were used to examine the relative microbial abundance. Based on One Codex Library, the Chao1, Simpson and Shannon indexes were calculated to evaluate the alpha diversity. The principal coordinate analysis (PCoA) was conducted to assess the beta diversity according to Bray-Curtis distance matrix. Permutational multivariate analysis of variance (PERMANOVA) was used to examine the statistical differences among groups. Additionally, the differential bacterial taxa of various groups at all taxonomic levels were identified using the linear discriminant analysis effect size (LEfSe) tool^4^. To deeply investigate the enhanced metabolic pathways, the HMP Unified Metabolic Analysis Network 3 (HUMAnN3) was utilized based on One Codex database^5^. Gene families identified by UniRef90 database using Diamond were mapped to KEGG orthology database and analyzed with LEfSe tool. For data visualization, cluster heatmap, PCoA plot, cladogram and bar plots of LEfSe results were generated using OmicStudio tools^6^.

**Supplementary References**

1. Zudaire E, Gambardella L, Kurcz C, Vermeren S. A computational tool for quantitative analysis of vascular networks. *PLoS One* 2011, **6**(11)**:** e27385.

2. Ames SK, Hysom DA, Gardner SN, Lloyd GS, Gokhale MB, Allen JE. Scalable metagenomic taxonomy classification using a reference genome database. *Bioinformatics* 2013, **29**(18)**:** 2253-2260.

3. Wood DE, Salzberg SL. Kraken: ultrafast metagenomic sequence classification using exact alignments. *Genome Biol* 2014, **15**(3)**:** R46.

4. Segata N, Izard J, Waldron L, Gevers D, Miropolsky L, Garrett WS*, et al.* Metagenomic biomarker discovery and explanation. *Genome Biol* 2011, **12**(6)**:** R60.

5. Franzosa EA, McIver LJ, Rahnavard G, Thompson LR, Schirmer M, Weingart G*, et al.* Species-level functional profiling of metagenomes and metatranscriptomes. 2018, **15**(11)**:** 962-968.

6. Lyu F, Han F, Ge C, Mao W, Chen L, Hu H*, et al.* OmicStudio: A composable bioinformatics cloud platform with real-time feedback that can generate high-quality graphs for publication. 2023, **2**(1)**:** e85.

**Supplementary tables**

**Table S1.** Sequences of primers for mouse quantitative real-time PCR.

| **Gene** | **Forward primer (5’-3’)** | **Reverse primer (5’-3’)** |
| --- | --- | --- |
| *Hand-1* | ATCATCACCACTCACACCCG | CTCTGGAAGTAAGGCCGCTC |
| *Prl2c2* | AGCCAGGCTCACACACTATT | ACTAGATCGTCCAGAGGGCT |
| *Prl3d1* | GGCCGCAGATGTGTATAGGG | AGTTTCGTGGACTTCCTCTCG |
| *Ascl2* | GTGAAGGTGCAAACGTCCAC | CCCTGCTACGAGTTCTGGTG |
| *Tpbpa* | GCCAGTTGTTGATGACCCTGA | GCTGTCCATGTTACTGTGGCT |
| *Junb* | AGGCAGCTACTTTTCGGGTC | TTGCTGTTGGGGACGATCAA |
| *Gab1* | ATTTCCACCGTGGATTTGAAC | GATCTATCGCTCGGAAAGGTC |
| *Gcm1* | AAGCTTATTCCCTGCCGAGG | AAAGATGAAGCGTCCGTCGT |
| *Gapdh* | TGACGTGCCGCCTGGAGAAA | AGTGTAGCCCAAGATGCCCTTCAG |

**Table S2.** Overview of primers and conditions for the pyrosequencing.

| ***Igf2*** | | |  |  |
| --- | --- | --- | --- | --- |
| Primer sequence | | | | PCR program (45 cycles) |
| Fwd | 5’-GGGTTGGGGTGGTTATTTTAATGG-3’ | | | 15 min 95°C  30 sec 94°C  60 sec 56°C  45 sec 72°C  7 min 72°C |
| Rev | 5'-gacgggacaccgctgatcgtttaATCTCTTTATCTCACCCCATAATTC-3' | | |  |
| UniRev | 5'-Biotin-gggacaccgctgatcgttta-3' | | |  |
| Seq | 5'-GAATTTTTAGGTAGGTTTTTAAG-3' | | |  |
| **Seq to analyze** | | | TYGTGTTAATYGTYGTAGTYGTGGTATYGTGGAAGAGTGTTGTTTT | |
| ***Vegf*** | | |  | PCR program (45 cycles) |
| Primer sequence | | | | 15 min 95°C  30 sec 94°C  30 sec 54°C  30 sec 72°C  7 min 72°C |
| Fwd | 5'-GAGTAAGAGTTGGGGTGTGTATAATG-3' | | |  |
| Rev | 5'-Biotin-AAACAACTAAAATAACCCCTAACTTTC-3' | | |  |
| Seq | 5'-GGTGTGTATAATGTAGTTATTAG-3' | | |  |
| **Seq to analyze** | | GGGGYGTTYGGTTATTAYGGGGAGATYGTGAATTTGGGYGAGTYGAGTTTGYGTGAGGGAGGAYGYGTGTGTTAATGTGAGTG | | |

**Supplementary Figures**

**
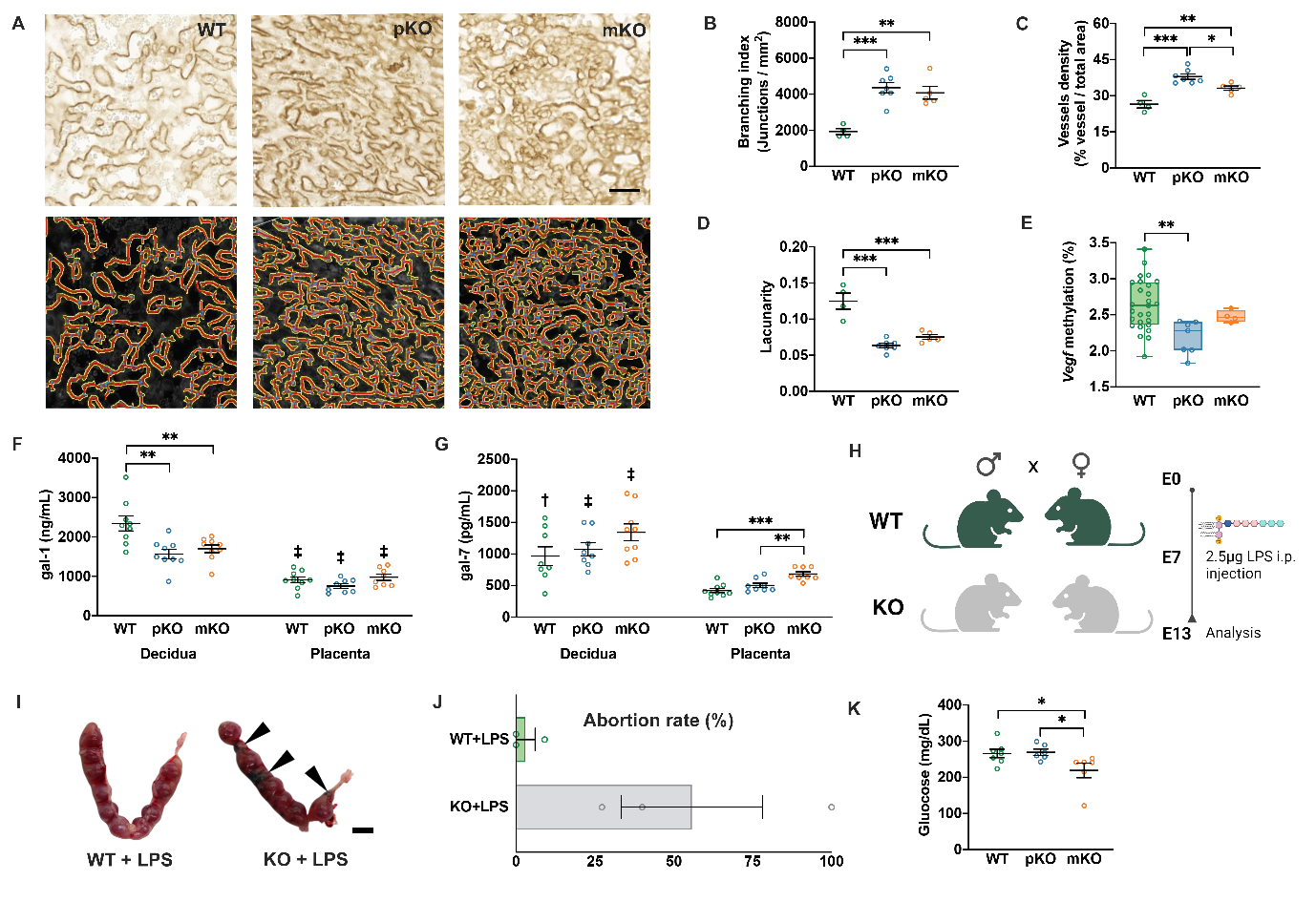
**

**Suppl. Fig. 1: A** Isolectin B4 (IB4) staining was performed to visualize the vascular network in the labyrinth (Lab) layer of E13 placenta (upper panel, scale bar = 50 µm). Inverted representative images were created by AngioTool software with the skeletonized vessels in red and branching points (junctions) in blue (lower panel). **B-D** Quantitative results of AngioTool analysis regarding the branching index, vessel density, and lacunarity (n=4-7). **E** Methylation levels of *Vegf* was decreased in gal-3 pKO placentas compared to the WT counterparts (n=4-27). **F** The expression of gal-1 in the decidua and placenta was measured by ELISA on E13 (n=8-9). **G** Decidual and placental gal-7 expression was measured by ELISA on E13 (n=8-9). **H** Schematic diagram of lipopolysaccharide (LPS) intraperitoneal (i.p.) injection on gal-3 WT (Lgals3^+/+^) and gal-3 KO (Lgals3^-/-^) females on E7. **I** Representative photographs of whole implantation sites upon LPS treatment on E13. Black arrows pointed to fetal resorptions (scale bar = 1.0 cm). **J** The abortion rate was calculated on E13 as (number of resorptions x 100) / total number of implantations (n=3-4). **K** Maternal glucose levels on E13 were measured by ELISA. In all scatter plots, data were presented as the mean ± SEM. In box and whisker plots, data were presented as minimum, lower quartile, median, upper quartile and maximum. **P < 0.05,* ***P < 0.01* and ****P < 0.001* using one-way ANOVA followed by Tukey's multiple comparisons test or Kruskal-Wallis test followed by Dunn's multiple comparisons test. *† P < 0.01* and *‡ P < 0.001* using unpaired Student's t test to assess the decidual and placental gal-1 or gal-7 expression levels within the group.

**
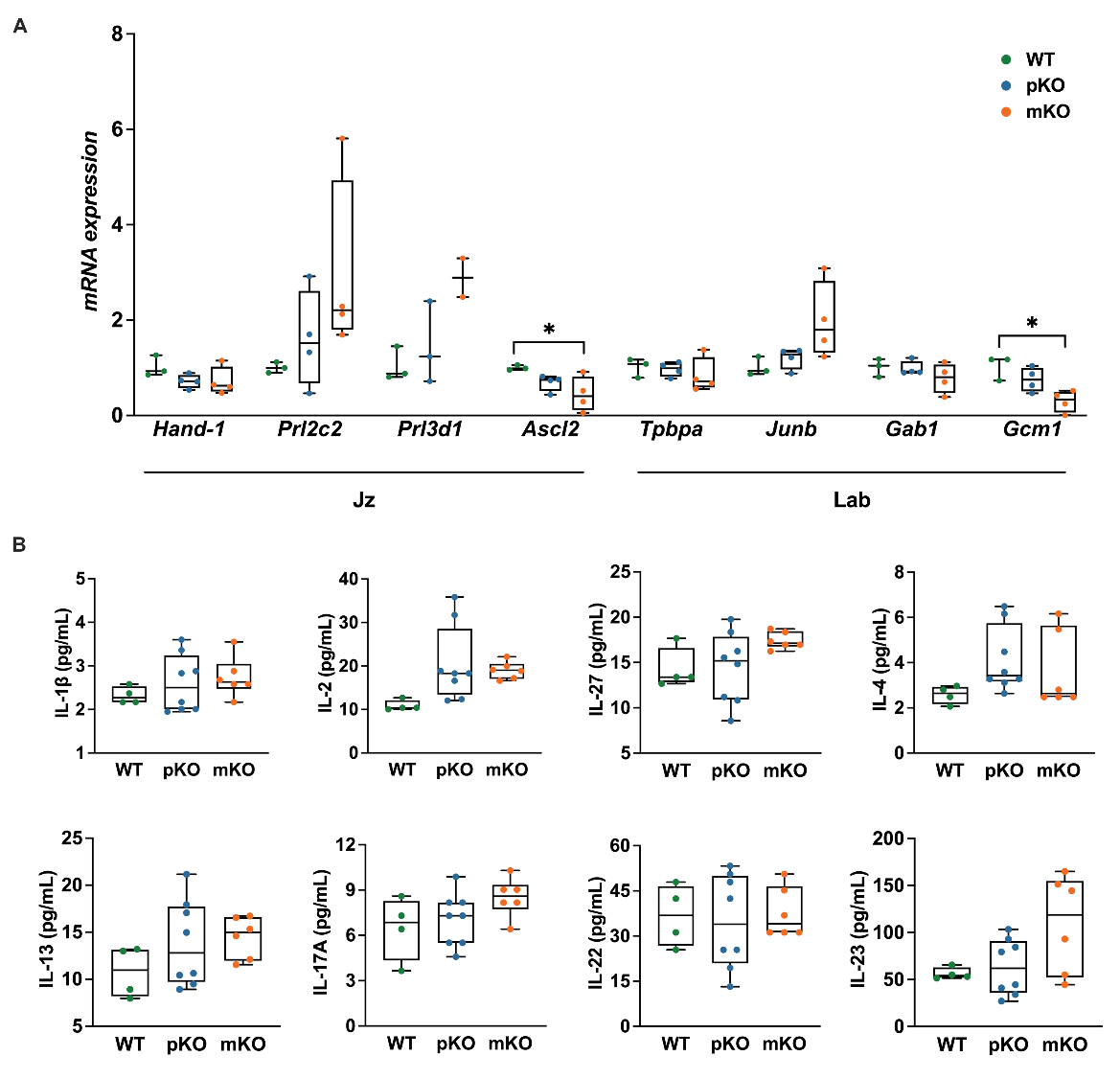
**

**Suppl. Fig. 2:** **A** The relative mRNA expression levels of trophoblast differentiation genes on E13 placentas involved in the junctional zone (Jz) or labyrinth (Lab) lineages were visualized as box plot (n=3-4). **P < 0.05* using one-way ANOVA followed by Tukey's multiple comparisons test. **B** Placental cytokines signature was determined by Luminex and represented with dotted box plots (n=4-6).

**
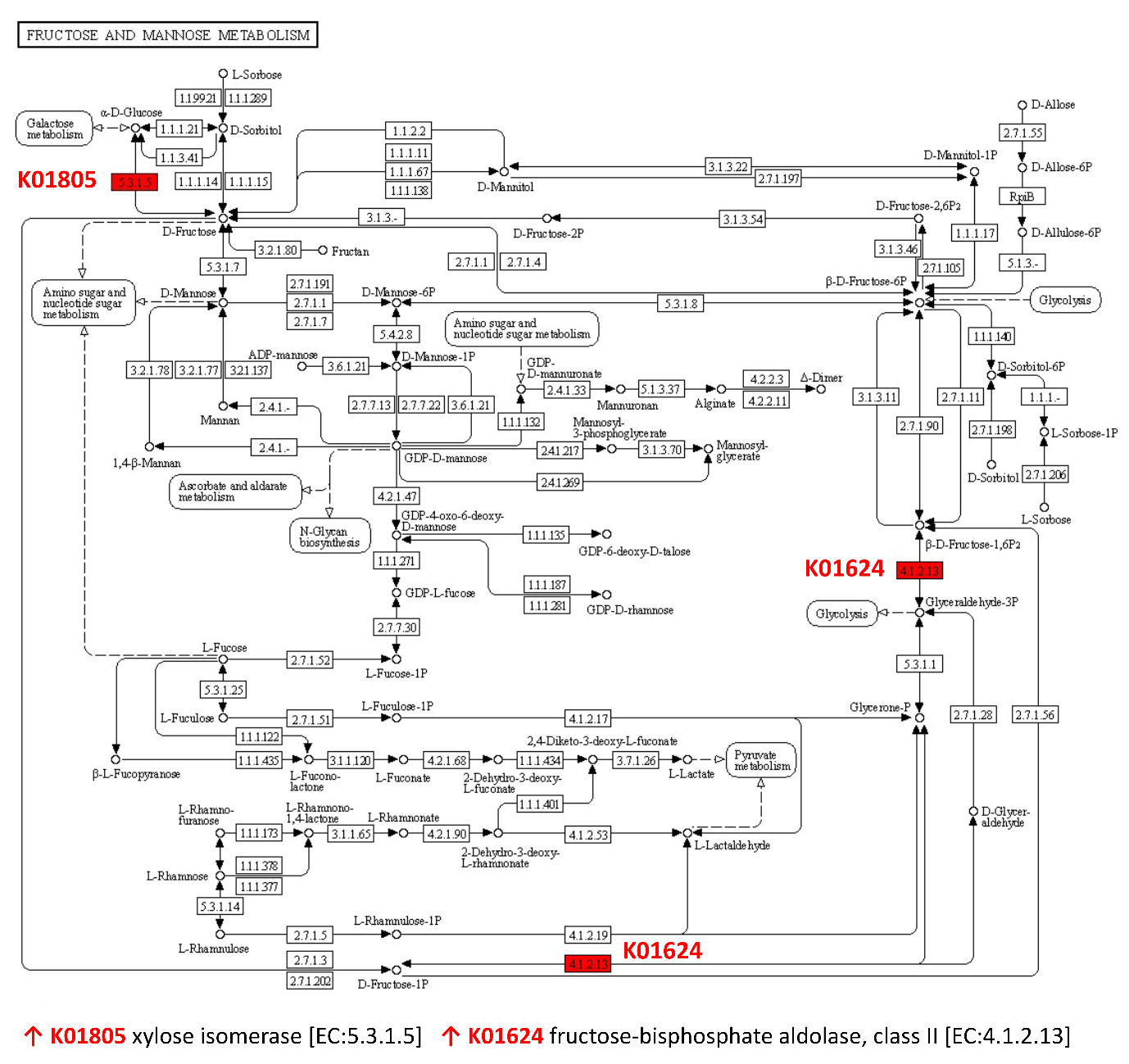
**

**Suppl. Fig. 3:** Enriched KEGG orthology terms in the gut microbiota of gal-3 mKO dams were involved in the fructose and mannose metabolism (Red represents upregulated KEGG orthology terms)
